# A taxonomically reliable DNA barcode reference library for North Sea macrobenthos

**DOI:** 10.1038/s41597-025-05500-z

**Published:** 2025-07-11

**Authors:** Magdalini Christodoulou, Sofie Derycke, Kevin K. Beentjes, Hans Hillewaert, Silke Laakmann, Kennet Lundin, Elham Kamyab, Sahar Khodami, Sara Maes, Henning Reiss, Carolin Uhlir, Laure Van den Bulcke, Berry Van der Hoorn, Annelies De Backer, Pedro Martinez Arbizu

**Affiliations:** 1 OÖ Landes-Kultur GmbH, Biodiversity Center, Linz, 4040 Austria; 2 Senckenberg am Meer, German Centre for Marine Biodiversity Research (DZMB), Wilhelmshaven, 26382 Germany; 3 Flanders Research Institute for Agriculture, Fisheries and Food – Marine Research (ILVO), Oostende, 8400 Belgium; 4 Naturalis Biodiversity Center, Leiden, 2333 CR The Netherlands; 5 Helmoltz Institute for Functional Marine Biodiversity at the University of Oldenburg (HIFMB), Oldenburg, 26129 Germany; 6 Alfred-Wegener-Institute (AWI), Helmholtz Centre for Polar and Marine Research 27570, Bremerhaven, Germany; 7 Gothenburg Natural History Museum, Gothenburg, S-40235 Sweden; 8 Faculty of Biosciences and Aquaculture, Nord University, Bodø, 8026 Norway; 9 Inholland University of Applied Sciences, AL Delft, 2628 The Netherlands

**Keywords:** Ecological genetics, Biodiversity, Taxonomy

## Abstract

EU directives (e.g. MSFD, Habitats Directive), along with OSPAR guidelines, mandate sustainable marine resource management across national borders. Benthic organisms are crucial for assessing marine ecosystem health, but their morphological identification is time-consuming and costly. High-throughput sequencing, particularly DNA metabarcoding, offers an alternative. However, DNA-based monitoring requires substantial investment in high-quality DNA reference libraries. The GEANS project (Genetic Tools for Ecosystem Health Assessment in the North Sea Region) aimed to develop efficient DNA-based tools for benthic biomonitoring. GEANS created a curated DNA reference library (COI) for species relevant to North Sea macrobenthos monitoring, using new sequences, non-public barcode sequences, and mined sequences from GenBank and BOLD. The library, stored in a dedicated BOLD project with photographs and metadata, includes DNA barcodes for 4005 specimens from 715 species, representing over 29% of North Sea macrobenthos species. Arthropoda is the most represented, while Bryozoa and Annelida have the lowest coverage. This DNA library is expected to facilitate fast, cost-effective environmental health assessments in the North Sea for public authorities and academics.

## Background & Summary

Macrobenthic invertebrates, animals larger than 1 mm, are key components in environmental monitoring and are extensively used for ecological status assessment of marine ecosystems worldwide because of their sensitivity to natural and anthropogenic disturbances^1–7^. Anthropogenic disturbances such as fisheries, sand extraction, pollution and shipping can impact growth, mortality, dispersal and recruitment of macrobenthic invertebrates, which in turn will affect ecosystem structure and function, along with their resilience^8–10^. Macrobenthic invertebrates are among the key obligatory components of biological monitoring surveys implemented in numerous countries in support of environmental directives, such as the European Union’s Marine Strategy Framework Directive (MSFD 2008/56/EC) and the Environmental Impact Assessment Directive (EIAD 2014/52/EU). Furthermore, for ecosystem health assessment, often ecological indices, such as the AZTI’s Marine Biotic Index (AMBI) and Shannon-Wiener diversity index (H’), are applied to macrobenthic communities, and these mostly require species-level identifications^2^.

Taxonomic identifications of macrobenthic invertebrates for routine assessments in marine areas, including the North Sea, have been carried out, using almost entirely morphology-based methodologies up to this day. This is a time- and cost-consuming, as well as a skill-dependent approach, which can result in a bottleneck (sampled vs processed specimens) in processing benthic samples for e.g. status assessments^11,12^. Moreover, species-level identifications can often be hindered, either because morphology-based identifications are difficult and require specialised expertise (especially true in groups such as Bryozoa, Hydrozoa, and Nemertea), or because during sampling and processing specimens get damaged and are missing key taxonomic characteristics. Furthermore, species-level identifications can be extremely difficult or nearly impossible when dealing with non-adult stages such as juveniles, larvae or eggs^12^. The increasing reports of cryptic species, even among many common macrobenthic species, complicates the morphology-based species identifications even further^13^. Finally, non-indigenous species resembling native species can be overlooked in routine rapid monitoring assessments^14^.

DNA-based approaches, such as DNA metabarcoding, have the potential to help tackle many of the limitations encountered by the morphology-based approach^15–19^. DNA metabarcoding appears to be more cost- and time-effective, does not require taxonomic experts for species identification and can detect non-indigenous, rare, or even undiscovered species that can go unnoticed with conventional methods^14,20,21^. Instead of the specimens being identified one by one morphologically, DNA is extracted from the total community, and a small fragment of the genome is amplified through PCR^22^. The resulting amplicons are sequenced using high-throughput sequencing and the sequences produced are processed through bioinformatic pipelines^23^. However, the potential power of DNA metabarcoding is currently limited mainly by the considerable endeavour needed to build comprehensive and reliable taxonomic sequence reference libraries that are required for matching DNA sequences to species names^15,24,25^. To ensure a high quality reference library, sequences must have *a priori* curated taxonomic information, and are preferably restricted to a list of species of the study area as taxonomic misassignment increases with geographic distance^25,26^. Most studies re-use sequences obtained from public sequence repositories, with the most common being NCBI GenBank (https://www.ncbi.nlm.nih.gov/genbank/)^27,28^ and Barcode of Life Data System (BOLD; http://www.boldsystems.org/)^29^. Although the databases’ importance is unquestionable, there is a significant percentage of sequence data without quality control and taxonomic validation that could lead to misleading results^25,30–32^.

**Fig. 1:**
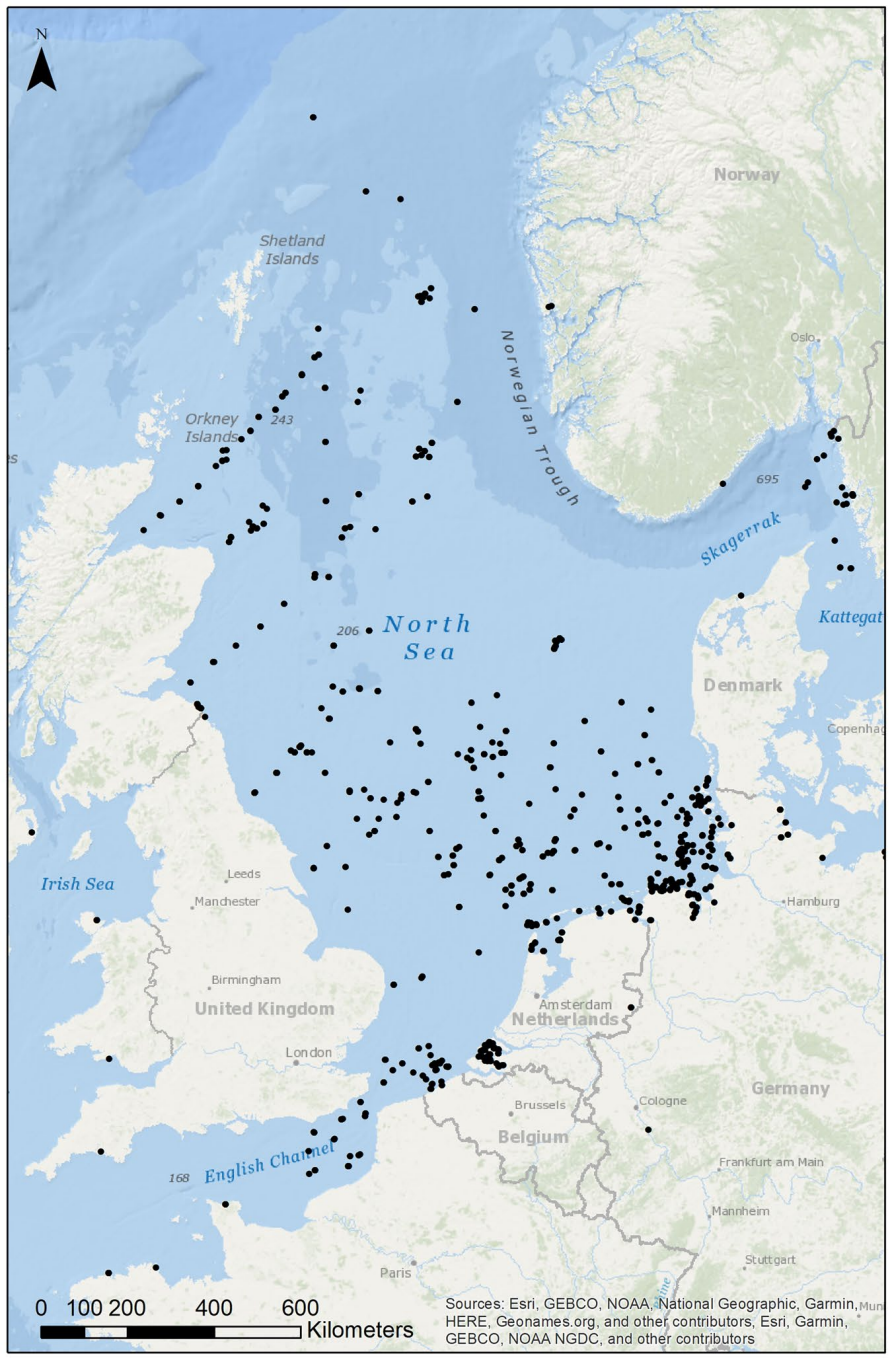
Map showing the collection localities of the barcoded specimens included in the GEANS reference library.

The North Sea is amongst the most heavily human-impacted marine areas worldwide^33–35^ (Fig. 1). At the same time, the North Sea is also one of the most well studied and data-rich marine areas in the world^34^, and it is routinely monitored by several countries organized in OSPAR (Convention for the Protection of the Marine Environment of the North-East Atlantic) and ICES (International Council for the Exploration of the Sea). The management of such a system with many anthropogenic pressures requires timely and efficient monitoring approaches. Therefore, the Interreg project GEANS (Genetic Tools for Ecosystem Health Assessment in the North Sea Region), a transnational project among nine institutions across the North Sea aimed to implement accurate, fast, cost-effective DNA-based tools in routine biomonitoring of the North Sea. To this end, GEANS deemed it essential to develop a curated DNA reference library based on mitochondrial cytochrome c oxidase subunit I (COI) for the North Sea macrobenthos (mainly soft bottom) in support of the routine monitoring programs in the North Sea. The choice of COI marker was driven by (i) the marker’s taxonomic resolution which permits species discrimination, identification and discovery in most of the marine invertebrate groups^15,36^; (ii) the vast amount of data already available as reference in the collaborators’ labs and in public repositories^27,29^ that could be used for cross-checking; (iii) the consistent use of COI in barcoding species and especially the 5′ end (COI*-*5P), the region that can be amplified using universal DNA-barcoding primers, such as LCO1490/HCO2198^37^ and their variations developed in recent years^38^. Furthermore, prior national and international initiatives have demonstrated the general effectiveness of DNA barcoding for various marine invertebrate groups in the North Sea, such as Mollusca^39^, Echinodermata^40^, Crustacea^41,42^.

**Fig. 2:**
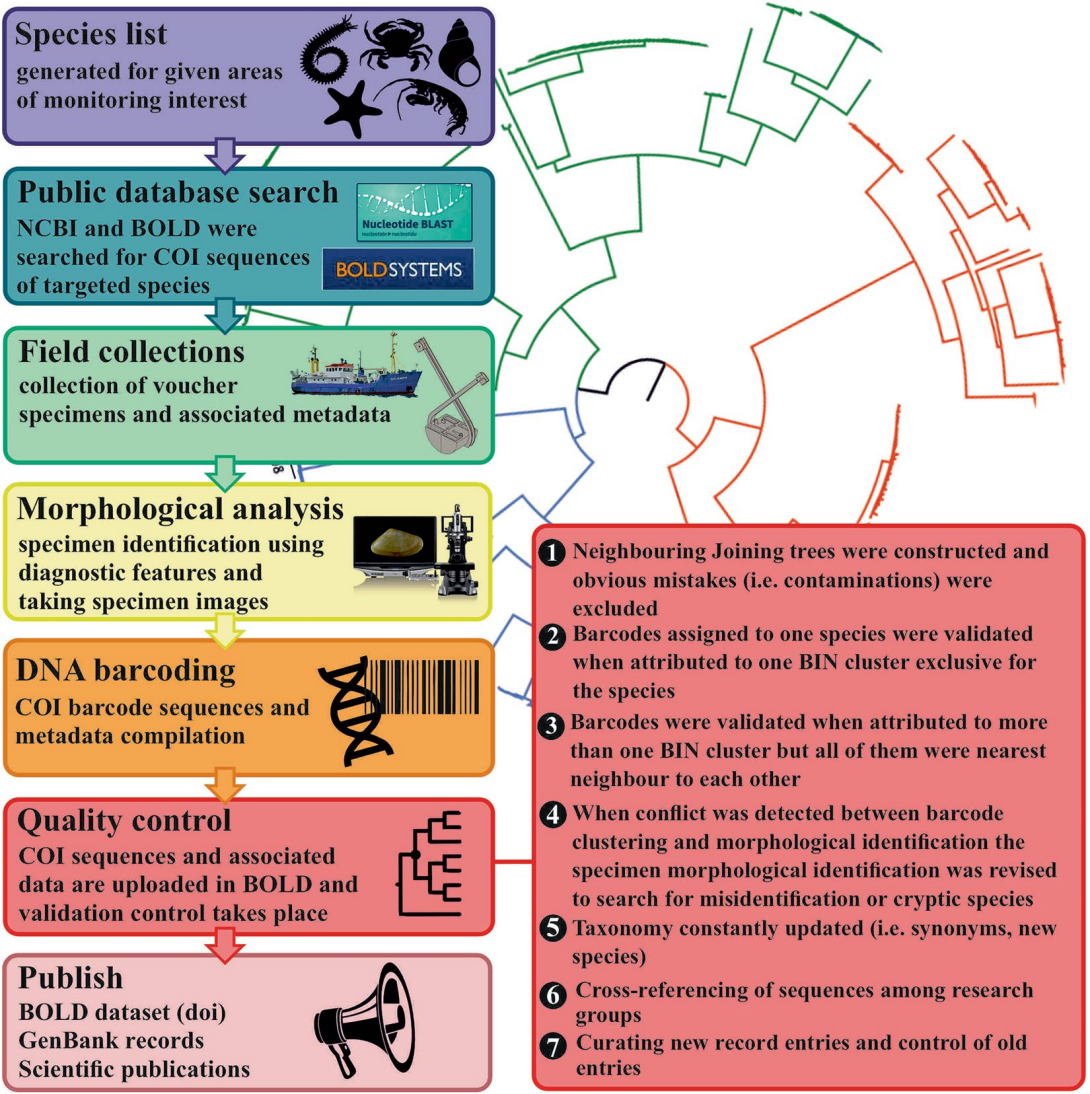
Simplified overview of curation workflow for the GEANS reference library. Inspired by Collins *et al*. 2021 (logos and images are public domain and were acquired from https://www.phylopic.org, https://www.ncbi.nlm.nih.gov, https://boldsystems.org, https://www.keyence.eu, https://www.deutsche-meeresforschung.de).

The aim of the present work was to create a curated DNA barcode reference library for the macrobenthic invertebrates of the North Sea (with priority to soft bottoms) by: (i) producing new COI reference sequences from a targeted region-defined species list; (ii) assessing and curating COI data already available to the collaborators’ labs; and (iii) providing a workflow for the creation of a curated reference library (Fig. 2).

**Fig. 3:**
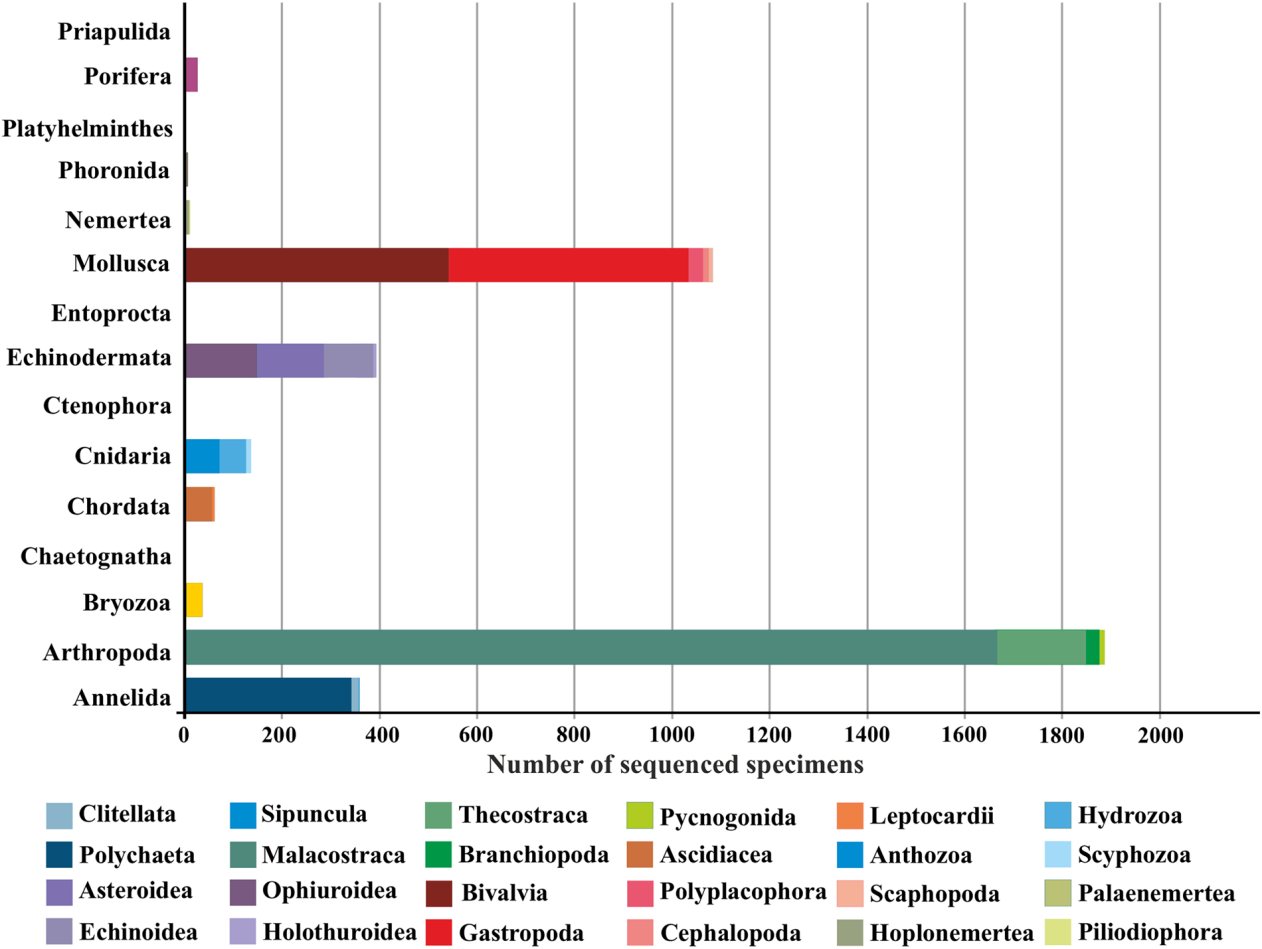
Taxonomic composition of the 4005 sequenced invertebrate marine specimens included in the GEANS DNA reference library (ds-GEANS1).

**Fig. 4:**
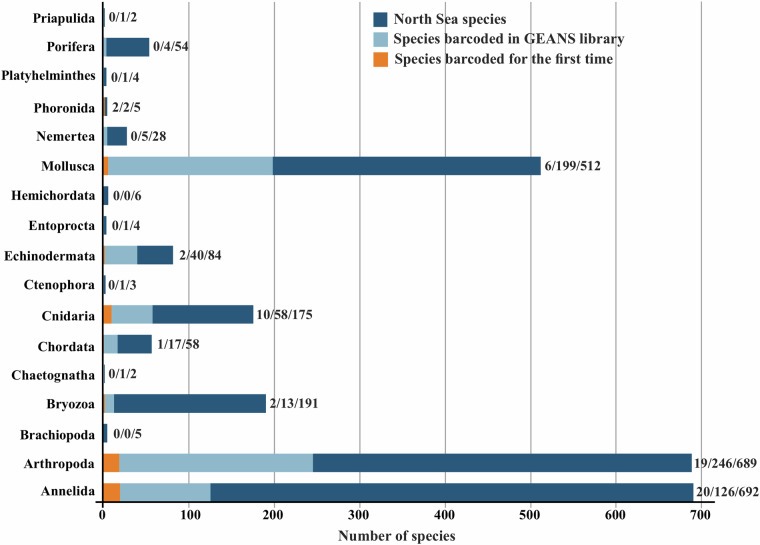
Barcode coverage of marine macrobenthic species of the North Sea in the GEANS DNA reference library. Numbers on bars refer to the species barcoded in comparison to the North Sea species.

**Fig. 5:**
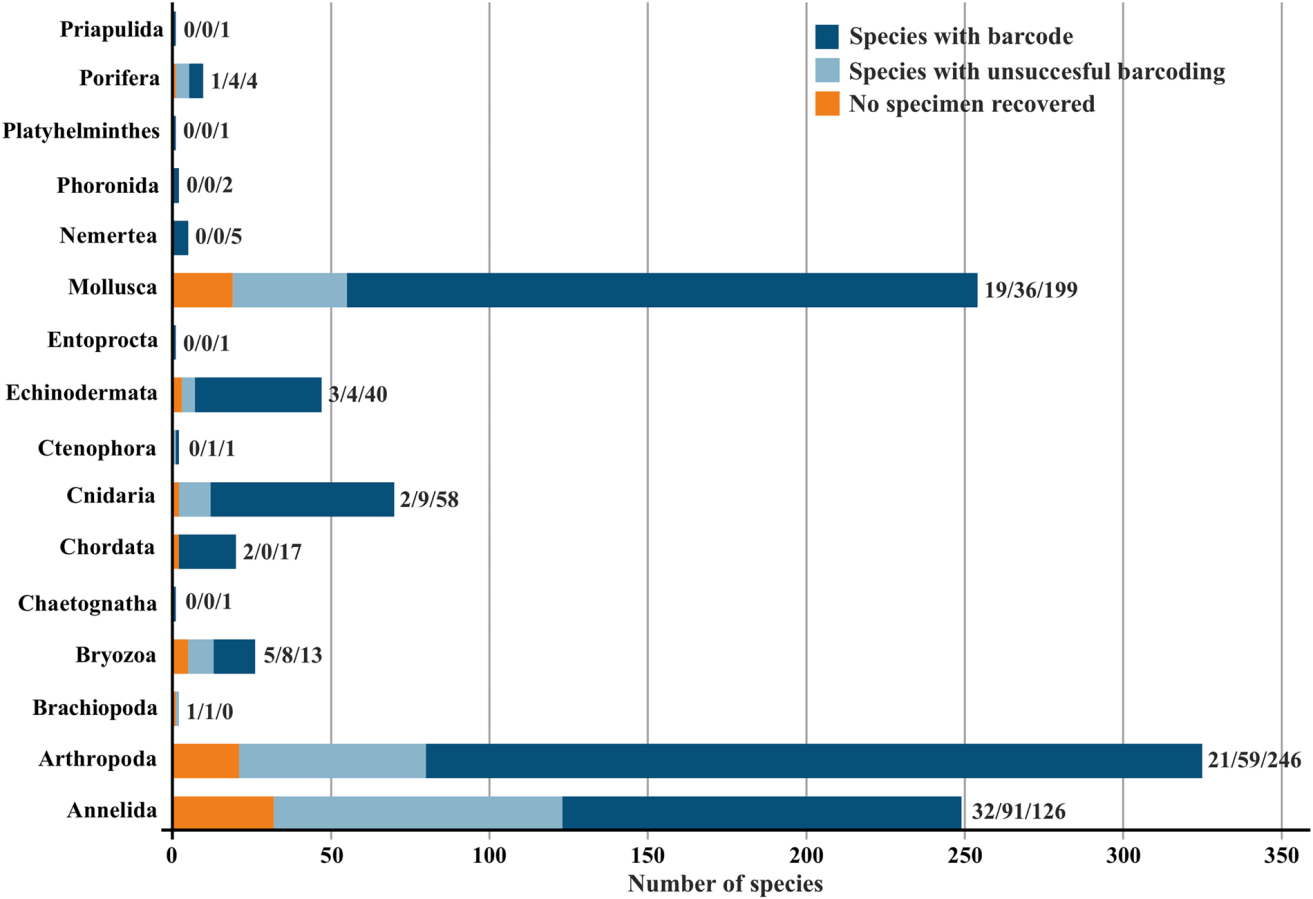
Barcode coverage of marine macrobenthic species of the North Sea included in the GEANS target list. Numbers on bars refer to the species successfully barcoded, species with specimens present but with unsuccesfully barcoding, and to species with no specimens aquired.

The GEANS reference library numbers a total of 4005 COI-5P barcode sequences from 732 (715 identified to species level) macrobenthic taxa and assigned to 764 BINs (Barcode Index Number^43^), which in turn were distributed over 15 phyla, 29 classes, 92 orders, 333 families, and 537 genera (Fig. 3; GEANS Reference Library^44^). The reference library, when compared to the number of macrobenthic species (2514 species, North Sea species list^44^) present in the North Sea covers over 29% of macrobenthos species diversity. Of the total number of taxa barcoded and identified to species level (715), 77 correspond to NIS (GEANS targeted species list^44^). A total of 1714 new DNA barcodes were generated through this study, of which 173 belonged to 62 species barcoded for the first time (Fig. 4; Table 1). The number of individuals per species ranged from 1 to 158, with 346 species (48%) represented by less than three individuals, 272 of which were represented by only a single specimen. Arthropoda was the most well represented taxon in number of sequences in the library with 1886 (47%; Fig. 3; Table 1) belonging to 246 species (Figs. 4, 5). Annelida, although recorded by a low number of sequences (358), were well represented in the reference library (126 species, 18% of the total number of species barcoded within GEANS, Fig. 5). Among all sequenced groups, Echinodermata had the highest barcode coverage with 93% (corresponding to 40 species; Fig. 5) of all Echinodermata species included in the GEANS target checklist being barcoded and 48% (40 species) when compared to the whole North Sea fauna (84 species; Fig. 4). In contrast, Bryozoa had the lowest barcode coverage with 50% of the total number of Bryozoa species in the checklist but only 8% when compared with the total North Sea fauna (Figs. 4, 5).

**Table 1:** Number of sequenced specimens, genera, species and the corresponding BINs per phylum included in the GEANS reference library.

Phylum	Collected species	Barcoded specimens	Barcoded genera	Barcoded species	BINs
Annelida	217	358 (312)	95	126/**20**	133
Arthropoda	304	1886 (562)	158	246/**19**	268
Brachiopoda	1	0	0	0	0
Bryozoa	21	35 (33)	12	13/**2**	15
Chaetognatha	1	1 (1)	1	1/**0**	1
Chordata	18	61 (56)	15	17/**1**	22
Cnidaria	67	138 (129)	53	58/**10**	58
Ctenophora	2	1 (0)	1	1/**0**	1
Echinodermata	44	394 (78)	34	40/**2**	44
Entoprocta	1	1 (1)	1	1/**0**	1
Mollusca	235	1083 (497)	154	199/**6**	204
Nemertea	5	11 (11)	6	5/**0**	5
Phoronida	2	6 (6)	1	2/**2**	2
Platyhelminthes	2	2 (2)	1	1/**0**	1
Porifera	9	27 (26)	4	4/**0**	8
Priapulida	1	1 (1)	1	1/**0**	1
**Total number**	**930**	**4005 (1714)**	**537**	**715**/**62**	**764**

In the parenthesis are given the number of specimens barcoded during the GEANS project, while the number of species barcoded for the first time per phylum are indicated in bold.

## Methods

The curated DNA barcode reference library (GEANS Reference Library^44^) presented here for North Sea macrobenthos was constructed based on a seven-step workflow (Fig. 2) that generated a diverse set of validated data starting with a targeted species checklist (GEANS targeted species list) restricted mainly to the south North Sea (Fig. 1).

### Targeted species checklist and North Sea species list

The nine GEANS partners (originating from seven countries: Belgium, Denmark, Germany, Netherlands, Norway, Sweden, United Kingdom) provided regional species lists based on species encountered in their long-term morphology-based monitoring data. This resulted in a concatenated target list (GEANS targeted species list) of 1016 marine macrobenthic species (119 non-indigenous species, NIS), that were checked for taxonomy (e.g. synonyms removed, checking validity of species names). As such, the majority of these species occur in areas of the North Sea, where the GEANS partners performed the case studies for testing the effectiveness of metabarcoding for specific monitoring questions^14,45,46^. The targeted checklist served as the basis of the GEANS reference library. To put the targeted list in a wider North Sea perspective, a North Sea macrobenthic species list^44^ was generated. This North Sea species list was created after extracting macrobenthic data from EurOBIS in a similar manner as Herman *et al*.^47^. Additionally, it was completed by the list of Zettler *et al*.^48^, and cross-checked by the list provided by WORMS for the North Sea which in turn was verified based on the relevant literature.

### Specimen collection, and identification of specimens

Specimens were collected from the North Sea during various research expeditions that took place in the years 2019–2021 using Van Veen grabs, boxcorers, and dredges (ring dredge, Triple-D dredge). Sampling was conducted by three GEANS partners, the German Centre for Marine Biodiversity Research (DZMB), Naturalis Biodiversity Center (Naturalis), and Flanders Research Institute for Agriculture, Fisheries and Food (ILVO) with research vessels RV Senckenberg, RV Pelagia, RV Belgica A956, RV Simon Stevin and GeoSurveyor XI. Subsequently, the same partners performed the morphological and genetic analyses. Following their collection, bulk samples or separated animals were fixed in precooled 96% or 99.8% ethanol. For all samples and specimens, DZMB collected, the ethanol was decanted after 24 hours and replaced with new 96–99.8% EtOH to guarantee sufficient ethanol concentration for preservation of high-quality DNA, and subsequently stored at −20 °C in one or more of the collaborative laboratories. In the laboratory, samples were sorted and identified at species level by taxonomic experts. The taxonomic status of each species was validated based on the World Register of Marine Species (www.marinespecies.org). For each species, when possible, at least three voucher specimens were archived. Additional specimens were provided by the Gothenburg Natural History Museum, Sweden as well as by the German authority Landesbetrieb für Küstenschutz Nationalpark und Meeresschutz Schleswig-Holstein.

### Barcoding data collection

#### DNA extraction, amplification, and sequencing

Total genomic DNA was extracted from animal tissue. At DZMB for samples where DNA of high quality was expected, the DNA extractions were carried out using 30 μL Chelex (InstaGene Matrix, Bio-Rad) according to the protocol of Estoup *et al*.^49^ and directly using it as DNA template for PCR. For samples where DNA of low quality was expected the Monarch Genomic DNA Purification Kit was used following manufacturer’s instructions. At ILVO, DNA was extracted using the DNeasy Blood & Tissue kit (Qiagen) following the manufacturer’s protocol. The concentration of the DNA was determined using the Quantus Fluorometer with the QuantiFluor dsDNA System (Promega). At Naturalis, DNA was extracted using the NucleoMag 96 Tissue kit (Macherey-Nagel) on the KingFisher (Thermo Scientific) according to the manufacturer’s protocol. DNA extractions were stored at −20 °C. A fragment of 658 bp of the mitochondrial cytochrome c oxidase subunit (COI), which is the standard barcoding marker for animals, was amplified by polymerase chain reaction (PCR). Amplifications in DZMB were performed using AccuStart PCR SuperMix (ThermoFisher Scientific) in a 25-μL volume using a standardised protocol (Table 2). All PCR products were purified using ExoSap-IT (ThermoFisher Scientific). Amplifications in Naturalis were performed using Phire II Hotstart (Thermo Scientific) in a 25 μL volume (Table 2). For the COI amplification the degenerate forward primers jgLCO1490 and reverse primer jgHCO2198^38^, tailed with M13F and M13R-pUC, respectively, were used both by DZMB and Naturalis. DZMB also used the Echinodermata specific forward primer, LCOech1aF1^50^, a polychaeta specific primer pair^51^ whereas, a universal pair that amplifies a shorter barcode region was also tested^11^. Amplifications in ILVO were performed with LCO1490 and HCO2198 primers in a 40 μL volume (Table 2). PCR products produced by ILVO were purified using the Wizard® SV Gel and PCR Clean-Up System (Promega). Purified PCR products from SGN and ILVO were sequenced by Sanger sequencing in both directions at Macrogen Europe BV (Amsterdam, The Netherlands), whereas Naturalis fragments were sequenced at BaseClear BV (Leiden, The Netherlands).

**Table 2:** PCR amplification conditions for COI gene in each research institute.

PCR recipe (μL)		DZMB		Naturalis		ILVO	
template DNA		2		2		2	
Master Mix		12.5*		6**		20***	
Forward primer, 10 pmol/μL		0.5		1.3		4	
Reverse primer, 10 pmol/μL		0.5		1.3		4	
ddH2O		to 25		to 25		to 40	
**PCR program (steps)**		**T, °C**	**Time**	**T, °C**	**Time**	**T, °C**	**Time**
Initial denaturation		94	3′	98	30″	94	2′
35–40 cycles	Denaturation	94	30″	98	5″	94	1′
	Annealing	47	60″	50	5″	50	1′
	Elongation	72	1′	72	15″	72	1′
Final elongation		72	5′	72	5′	72	7′

*AccuStart PCR SuperMix (ThermoFisher Scientific).

**0.5 μL Phire II Hotstart polymerase, 5.0 μL Phire buffer, 0.5 µL dNTP (2.5 mM).

***20 µl Red Taq DNA-polymerase: 2x MasterMix, 1,5 mM MgCl_2_ (VWR).

#### Existing barcodes in collaborators databases

The collaborators’ internal databases were mined for barcode sequences of macrobenthic animals collected from the North Sea. Only barcodes above 500 bp were considered, unless shorter fragments were the only ones available for a targeted species. Specifically, the DZMB completed the GEANS reference library with COI sequences from past barcode initiatives such as the “Molecular taxonomy and DNA barcoding of marine organisms (metazoa) of the North Sea”^39–42^. These sequences correspond to specimens or tissue archived in DZMB’s collections. The forward and reverse sequence chromatograms for each specimen were inspected, assembled, and edited using Geneious v.9.1.7 (www.geneious.com^52^). The COI sequences were aligned using MAFFT v7.308^53^ under G-INS-I algorithm, while alignments were further manually edited.

## Data Records

**Fig. 6:**
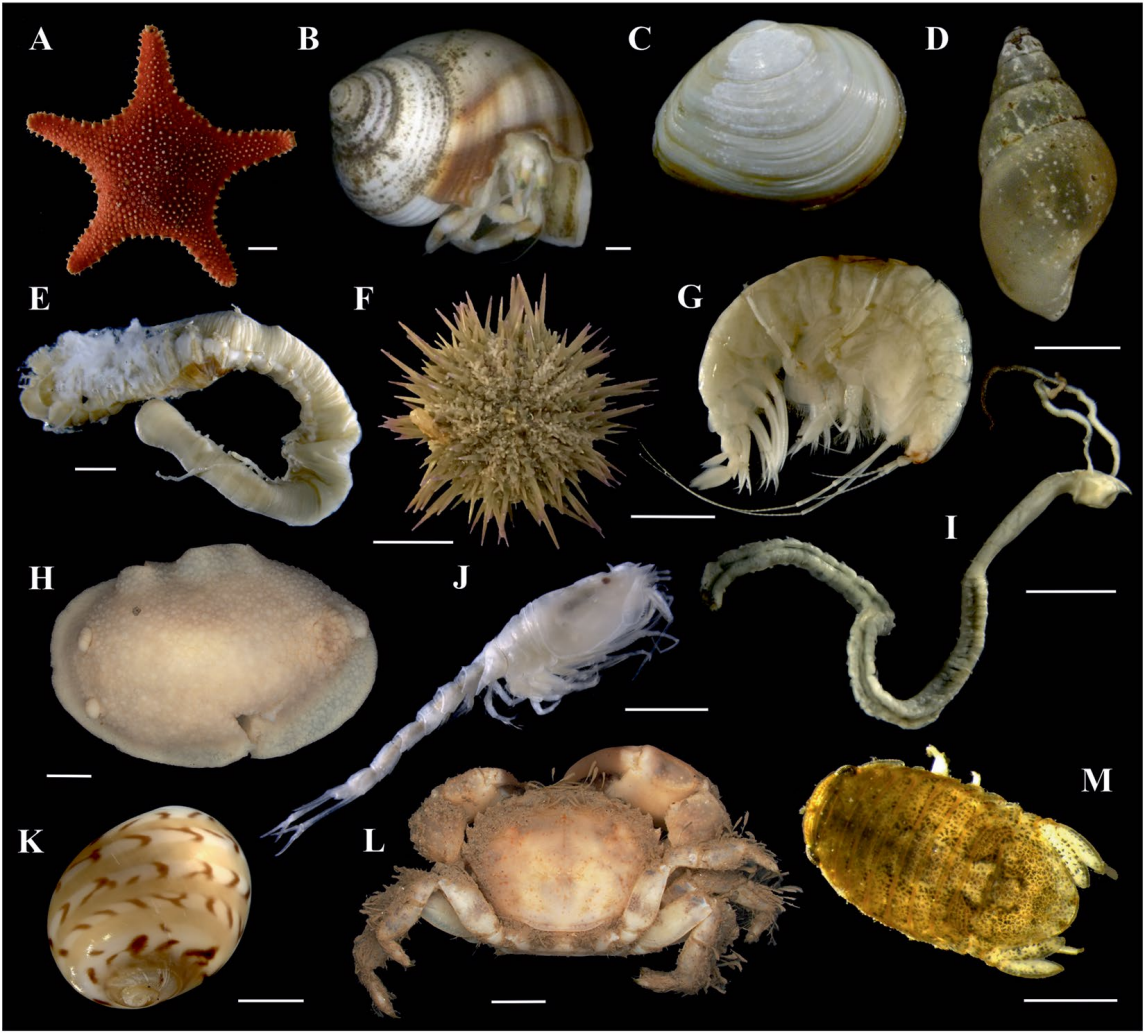
GEANS reference library online gallery of photo vouchers of sequenced specimens identified by taxonomic experts. (**A**) *Hippasteria phrygiana* (Parelius, 1768); (**B**) *Pagurus bernhardus* (Linnaeus, 1758); (**C**) *Macoma balthica* (Linnaeus, 1758); (**D**) *Peringia ulvae* (Pennant, 1777); (**E**) *Loimia ramzega* Lavesque *et al*. 2017; (**F**) *Psammechinus miliaris* (P.L.S. Müller, 1771); (**G**) *Ampelisca brevicornis* (A. Costa, 1853); (**H**) *Doris pseudoargus* Rapp, 1827; (**J**) *Diastylis bradyi* Norman, 1879; (**I**) *Magelona johnstoni* Fiege, *et al*., 2000; (**K**) *Euspira nitida* (Donovan, 1803); (**L**) *Pilumnus hirtellus* (Linnaeus, 1761); (**M**) *Lekanesphaera rugicauda* (Leach, 1814). Scales: 1 cm (**A, B, D, F**); 1 mm (**K,** **M**); 2 mm (**G, H, J, I, L**); 5 mm (**E**). Photos by: V. Borges (**A**); M. Christodoulou (**B, D, F**); H. Hillewaert (**C, E, G, J, I, K**); GiMaRIS (**H, L**); W. Stamerjohanns (**M**).

The GEANS Reference Library (summary information), GEANS Targeted Species List and North Sea species list and Neighbour Joining trees are available in Figshare^44^. Additionally in Figshare^44^ are found the DNA barcodes and specimen photos corresponding to the new barcodes produced. Additionally, barcodes produced during GEANS are available in GenBank (BioProject PRJNA1236822^54^). The data are available as well in BOLD through the dataset DS-GEANS1^55^ (dx.doi.org/10.5883/DS-GEANS1). Each COI barcode included in the GEANS reference library is accompanied by the following mandatory information: 1) sample ID; 2) specimen taxonomic identification and classification; 3) collection date; 4) collection coordinates; 5) storing institution; 6) when possible one photograph of the specimen (Fig. 6), when possible photos of the key diagnostic features; 7) name of taxonomic expert; 8) sequence chromatograms; 9) museum ID when specimens are archived in museum collections. Finally, the GEANS reference library also includes: (1) voucher specimens; (2) tissue samples; (3) total DNA extractions. A specimen was considered as a species reference when molecular and morphological assessments agreed. The library follows the barcode data standard requirements^29,32,36,56^. Samples and extractions are available in the partner institutes (DZMB, Naturalis, ILVO).

## Technical Validation

Each institution performed independent morphological identifications prior to the genetic identification. When disagreements were found, they were listed and the voucher specimens or the photos were revised to verify the original identifications. Obvious mistakes in identification or curation (e.g., mixing of photos for example) were corrected, in all other cases the mismatch between genetic and morphological identification was recorded as such. Finally, the species names were updated to the current taxonomy based on the World Register of Marine Species (WoRMS). Curation cycles were performed at regular intervals (Fig. 2). In addition to morphological validation, all barcodes were translated into amino acids to check for stop codons and to detect the presence of nuclear DNA pseudogenes (NUMTs). The obtained COI sequences were initially compared with the GenBank nucleotide database using BLASTN^57^ to confirm the phylum identity (Fig. 2). Additionally the BOLD database was used for verification once the barcodes were within, since BOLD contains more barcode sequences than GenBank (including unpublished barcodes). For each taxonomic group (phylum or order depending on the number of sequences), Neighbour Joining (NJ) analysis based on p-distances with 1000 non-parametric bootstrap replicates was performed using the software MEGA v.11^58^ and any irregularities (possible contaminations) were removed from the library (trees are available in Figshare^44^). Sequences were considered to be the same taxon if sequence identity was ≥97.5%.

## Usage Notes

The GEANS DNA reference library offers a comprehensive, publicly available barcode dataset for North Sea macrobenthos available in BOLD (DS-GEANS1 (dx.doi.org/10.5883/DS-GEANS1^55^). This resource enables specimen identification through barcoding and metabarcoding, thereby greatly facilitating macrobenthic biodiversity assessments using molecular tools in the North Sea region. The DNA barcode reference library presented in this study includes around 30% of North Sea macrobenthic species, and aims to complement and facilitate the morphological identification of species through barcoding or metabarcoding.

From the total number of targeted species in the checklist (1016 species, GEANS targeted species list), we were unable to recover barcode sequences for 215 species (21%), and were not successful in finding specimens for an additional 86 species (8%). The phylum with the lowest amplification success was Annelida-Polychaeta (37%, Fig. 5), followed by Arthropoda (18%) and Mollusca (14%).

The majority of BINs allocated to the species within the GEANS dataset were considered concordant (i.e., one BIN = one species) with 684 species corresponding to 96% of the total number of BINs (GEANS Reference Library). A total of 31 species were assigned to more than one BIN (72 BINs, 4% of the species). Although originally a larger number of BINs than the one mentioned above were found to be discordant (BINs shared by more than one species), a subsequent validation revealed that this was due mainly to misidentifications. A small number of shared BINs are most likely due to the presence of unvalidated or erroneously identified data in BOLD and not actually wrong records in our dataset, however some closely related species may not be distinguishable solely by the COI and they may appear sharing BINs. At the same time, a number of species found to hold more than one BIN could indicate the presence of cryptic species (e.g., *Astropecten irregularis, Crepidula fornicata, Hediste diversicolor*).

The library is expected to significantly expand the reach and accuracy of DNA metabarcoding studies in the North Sea whereas it allows for its continued growth to better understand the diversity of the North Sea fauna.

## Data Availability

No code was produced in the current study.

## References

[1] Dauer, D. M. Biological criteria, environmental health and estuarine macrobenthic community structure. *Mar. Pollut. Bull.* **26**, 249–257 (1993).

[2] Borja, A., Franco, J. & Pérez, V. A marine biotic index to establish the ecological quality of soft-bottom benthos within European estuarine and coastal environments. *Mar. Pollut. Bull.* **40**, 1100–1114 (2000).

[3] Rosenberg, R., Blomqvist, M., Nilsson, H. C., Cederwall, H. & Dimming, A. Marine quality assessment by use of benthic species-abundance distributions: a proposed new protocol within the European Union Water Framework Directive. *Mar. Pollut. Bull.* **49**, 728–739 (2004). 15530516 10.1016/j.marpolbul.2004.05.013

[4] Muxika, I., Borja, Á. & Bonne, W. The suitability of the marine biotic index (AMBI) to new impact sources along European coasts. *Ecol. Indic.* **5**, 19–31 (2005).

[5] Patrício, J., Neto, J. M., Teixeira, H., Salas, F. & Marques, J. C. The robustness of ecological indicators to detect long-term changes in the macrobenthos of estuarine systems. *Mar. Environ. Res.* **68**, 25–36 (2009). 19409610 10.1016/j.marenvres.2009.04.001

[6] Kröncke, I. *et al*. Changes in North Sea macrofauna communities and species distribution between 1986 and 2000. *Estuar. Coast. Shelf Sci.* **94**, 1–15 (2011).

[7] Coates, D. A., Deschutter, Y., Vincx, M. & Vanaverbeke, J. Enrichment and shifts in macrobenthic assemblages in an offshore wind farm area in the Belgian part of the North Sea. *Mar. Environ. Res.* **95**, 1–12 (2014). 24373388 10.1016/j.marenvres.2013.12.008

[8] Worm, B. *et al*. Impacts of biodiversity loss on ocean ecosystem services. *Science* **314**, 787–790 (2006). 17082450 10.1126/science.1132294

[9] Thrush, S. F. *et al*. Changes in the location of biodiversity–ecosystem function hot spots across the seafloor landscape with increasing sediment nutrient loading. *Proc. Biol. Soc.* **284**, 1471–2954 (2017). 10.1098/rspb.2016.2861 PMC5394661 28404774

[10] Lam-Gordillo, O., Baring, R. & Dittmann, S. Ecosystem functioning and functional approaches on marine macrobenthic fauna: A research synthesis towards a global consensus. *Ecol. Indic.* **115**, 106379 (2020).

[11] Lobo, J., Shokralla, S., Costa, M. H., Hajibabaei, M. & Costa, F. O. DNA metabarcoding for high-throughput monitoring of estuarine macrobenthic communities. *Sci. Rep.* **7**, 15618 (2017). 29142319 10.1038/s41598-017-15823-6 PMC5688171

[12] Hering, D. *et al*. Implementation options for DNA-based identification into ecological status assessment under the European Water Framework Directive. *Water Resour.* **138**, 192–205 (2018). 10.1016/j.watres.2018.03.003 29602086

[13] Grosse, M., Capa, M. & Bakken, T. Describing the hidden species diversity of *Chaetozone* (Annelida, Cirratulidae) in the Norwegian Sea using morphological and molecular diagnostics. *ZooKeys* **1039**, 139–176 (2021). 34113206 10.3897/zookeys.1039.61098 PMC8163715

[14] Hablützel, P. *et al*. DNA-based monitoring of Non-Indigenous Species. Pilot Report (Interreg North Sea Region GEANS, EU, 2023).

[15] Bucklin, A., Steinke, D. & Blanco-Bercial, L. DNA barcoding of marine metazoa. *Ann. Rev. Mar. Sci.* **3**, 471–508 (2011). 21329214 10.1146/annurev-marine-120308-080950

[16] Taberlet, P., Coissac, E., Pompanon, F., Brochmann, C. & Willerslev, E. Towards next‐generation biodiversity assessment using DNA metabarcoding. *Mol. Ecol.* **21**, 2045–2050 (2012). 22486824 10.1111/j.1365-294X.2012.05470.x

[17] Aylagas, E., Borja, A., Irigoien, X. & Rodriguez-Ezpeleta, N. Benchmarking DNA metabarcoding for biodiversity-based monitoring and assessment. *Front. Mar. Sci.* **3**, 96 (2016).

[18] Derycke, S. *et al*. Detection of macrobenthos species with metabarcoding is consistent in bulk DNA but dependent on body size and sclerotization in eDNA from the ethanol preservative. *Front. Mar. Sci*. **8** (2021).

[19] Van den Bulcke, L. *et al*. DNA metabarcoding on repeat: Sequencing data of marine macrobenthos are reproducible and robust across labs and protocols. *Ecol. Indic.* **150**, 110207 (2023).

[20] Rey, A., Basurko, O. C. & Rodriguez-Ezpeleta, N. Considerations for metabarcoding-based port biological baseline surveys aimed at marine nonindigenous species monitoring and risk assessments. *Ecol. Evol.* **10**, 2452–2465 (2020). 32184993 10.1002/ece3.6071 PMC7069299

[21] Bucklin, A. *et al*. Toward a global reference database of COI barcodes for marine zooplankton. *Mar. Biol.* **168**, 78 (2021).

[22] Leray, M. *et al*. A new versatile primer set targeting a short fragment of the mitochondrial COI region for metabarcoding metazoan diversity: application for characterizing coral reef fish gut contents. *Front. Zool.* **10**, 34 (2013). 23767809 10.1186/1742-9994-10-34 PMC3686579

[23] Pawloski, J. *et al*. The future of biotic indices in the ecogenomic era: Integrating (e)DNA metabarcoding in biological assessment of aquatic ecosystems. *Sci. Total Environ.* **637**, 1295–1310 (2018). 29801222 10.1016/j.scitotenv.2018.05.002

[24] Cristescu, M. E. & Hebert, P. D. N. Uses and misuses of environmental DNA in biodiversity science and conservation. *Annu. Rev. Ecol. Evol. Syst.* **49**, 209–230 (2018).

[25] Weigand, H. *et al*. DNA barcode reference libraries for the monitoring of aquatic biota in Europe: Gap-analysis and recommendations for future work. *Sci. Total Envir.* **678**, 499–524 (2019). 10.1016/j.scitotenv.2019.04.247 31077928

[26] Bergsten, J. *et al*. The effect of geographical scale of sampling on DNA barcoding. *Syst. Biol.* **61**(5), 851–69 (2012). 22398121 10.1093/sysbio/sys037 PMC3417044

[27] Clark, K., Karsch-Mizrachi, I., Lipman, D. J., Ostell, J. & Sayers, E. W. GenBank. *Nucleic Acids Res*. **44** (2016). 10.1093/nar/gkv1276 PMC4702903 26590407

[28] Leray, M., Knowlton, N., Ho, S. L., Nguyen, B. N. & Machida, R. J. GenBank is a reliable resource for 21st century biodiversity research. *Proc. Natl. Acad. Sci. USA* **116**(45), 22651–22656 (2019). 31636175 10.1073/pnas.1911714116 PMC6842603

[29] Ratnasingham, S. & Hebert, P. D. N. BOLD: The Barcode of Life Data System (www.barcodinglife.org). *Mol. Ecol. Notes* **7**, 355–364 (2007). 10.1111/j.1471-8286.2007.01678.x PMC1890991 18784790

[30] Locatelli, N. S., McIntyre, P. B., Therkildsen, N. O. & Baetscher, D. S. GenBank’s reliability is uncertain for biodiversity researchers seeking species-level assignment for eDNA. *Proc. Natl. Acad. Sci. USA* **117**, 32211–32212 (2020). 33234565 10.1073/pnas.2007421117 PMC7768753

[31] Collins, R. A. *et al*. Meta-Fish-Lib: A generalised, dynamic DNA reference library pipeline for metabarcoding of fishes. *J. Fish Biol.* **99**(4), 1446–1454 (2021). 34269417 10.1111/jfb.14852

[32] Pentinsaari, M., Ratnasingham, S., Miller, S. E. & Hebert, P. D. N. BOLD and GenBank revisited - Do identification errors arise in the lab or in the sequence libraries? *PLoS One* **15**(4), e0231814 (2020). 32298363 10.1371/journal.pone.0231814 PMC7162515

[33] Emeis, K. C. *et al*. The North Sea – A shelf sea in the Anthropocene. *J. Mar. Syst.* **141**, 18–33 (2015).

[34] Moullec, F. *et al*. Identifying and addressing the anthropogenic drivers of global change in the North Sea: a systematic map protocol. *Environ. Evid.* **10**, 19 (2021). 10.1186/s13750-025-00377-2 PMC12673759 41331668

[35] Daewel, U., Akhtar, N., Christiansen, N. & Schrum, C. Offshore wind farms are projected to impact primary production and bottom water deoxygenation in the North Sea. *Commun. Earth Environ.* **3**, 292 (2022).

[36] Hebert, P. D. N., Cywinska, A., Ball, S. L. & DeWaard, J. R. Biological identifications through DNA barcodes. *Proc. Roy. Soc. B: Biol. Sci.* **270**, 313–321 (2003). 10.1098/rspb.2002.2218 PMC1691236 12614582

[37] Folmer, O., Black, M., Hoeh, W., Lutz, R. & Vrijenhoek, R. DNA primers for amplification of mitochondrial cytochrome c oxidase subunit I from diverse metazoan invertebrates. *Mol. Mar. Biol. Biotechnol.* **3**(5), 294–299 (1994). 7881515

[38] Geller, J. B., Meyer, C. P., Parker, M. & Hawk, H. L. Redesign of PCR primers for mitochondrial Cytochrome c oxidase subunit I for marine invertebrates and application in all-taxa biotic surveys. *Mol. Ecol. Res.* **13**(5), 851–861 (2013). 10.1111/1755-0998.12138 23848937

[39] Barco, A., Raupach, M. J., Laakmann, S., Neumann, H. & Knebelsberger, T. Identification of North Sea molluscs with DNA barcoding. *Mol. Ecol. Resour.* **16**, 288–297 (2016). 26095230 10.1111/1755-0998.12440

[40] Laakman, S., Boos, K., Knebelsberger, T., Raupach, M. J. & Neumann, H. Species identification of echinoderms from the North Sea by combining morphology and molecular data. *Helg. Mar. Res.* **70**, 18 (2016).

[41] Raupach, M. J. *et al*. The application of DNA barcodes for the identification of marine crustaceans from the North Sea and adjacent regions. *PLoS ONE* **10**(9), e0139421 (2015). 26417993 10.1371/journal.pone.0139421 PMC4587929

[42] Beermann, J. *et al*. Cryptic species in a well-known habitat: applying taxonomics to the amphipod genus *Epimeria* (Crustacea, Peracarida). *Sci. Rep.* **8**, 6893 (2018). 29720606 10.1038/s41598-018-25225-x PMC5931980

[43] Ratnasingham, S. & Hebert, P. D. N. A DNA-based registry for all animal species: The Barcode Index Number (BIN) System. *PLoS ONE* **8**(7), e66213 (2013). 23861743 10.1371/journal.pone.0066213 PMC3704603

[44] Christodoulou, M. *et al*. A taxonomically reliable DNA barcode reference library for North Sea macrobenthos. 10.6084/m9.figshare.28408385. 10.1038/s41597-025-05500-z PMC12254315 40645994

[45] Obst, M. *et al*. Marine long-term biodiversity assessment suggests loss of rare species in the Skagerrak and Kattegat region. *Mar. Biodivers.* **48**, 2165–2176 (2017).

[46] Derycke, S. *et al*. DNA-based monitoring of soft sediments. Pilot 1 Report (Interreg North Sea Region GEANS, EU, 2023).

[47] Herman, P. M. J., Stolte, W. & van der Heijden, L. Summary presence/absence maps of macro-endobenthos in the greater North Sea, based of nearly 100,000 samples from 65 assembled monitoring data sets. *EMODNET biology data product*, https://www.emodnet-biology.eu/data-catalog?module=dataset&dasid=6617 (2020).

[48] Zettler, M. L. *et al*. An annotated checklist of macrozoobenthic species in German waters of the North and Baltic Seas. *Helgol. Mar. Res.* **72**, 5 (2018).

[49] Estoup, A., Largiader, C., Perrot, E. & Chouruout, D. Rapid one-tube DNA extraction for reliable PCR detection of fish polymorphic markers and transgenes. *Mol. Mar. Biol. Biotechnol.* **5**(4), 295–298 (1996).

[50] Layton, K. K. S., Corstorphine, E. A. & Hebert, P. D. N. Exploring Canadian echinoderm diversity through DNA Barcodes. *PLoS ONE* **11**(11), e0166118 (2016). 27870868 10.1371/journal.pone.0166118 PMC5117606

[51] Carr, C. M., Hardy, S. M., Brown, T. M., Macdonald, T. A. & Hebert, P. D. N. A Tri-Oceanic perspective: DNA barcoding reveals geographic structure and cryptic diversity in Canadian polychaetes. *PLoS ONE* **6**(7), e22232 (2011). 21829451 10.1371/journal.pone.0022232 PMC3136506

[52] Kearse, M. *et al*. Geneious Basic: an integrated and extendable desktop software platform for the organization and analysis of sequence data. *Bioinformatics* **28**, 1647–1649 (2012). 22543367 10.1093/bioinformatics/bts199 PMC3371832

[53] Katoh, K., Misawa, K., Kuma, K. I. & Miyata, T. MAFFT: a novel method for rapid multiple sequence alignment based 585 on fast Fourier transform. *Nucleic Acids Res.* **30**, 3059–3066 (2002). 12136088 10.1093/nar/gkf436 PMC135756

[54] Christodoulou, M. *et al*. *GenBank*. https://identifiers.org/ncbi/bioproject:PRJNA1236822.

[55] Christodoulou, M. *et al*. Dataset: DS-GEANS1. GEANS Reference. dx. 10.5883/DS-GEANS1 Library.

[56] Rimet, F. *et al*. Metadata standards and practical guidelines for specimen and DNA curation when building barcode reference libraries for aquatic life. *Metabarcoding Metagenom*. **5**, e58056, 17–33 (2021).

[57] Altschul, S. F., Gish, W., Miller, W., Myers, E. W. & Lipman, D. J. Basic local alignment search tool. *J. Mol. Biol.* **215**, 403–410 (1990). 2231712 10.1016/S0022-2836(05)80360-2

[58] Tamura, K., Stecher, G. & Kumar, S. MEGA11: Molecular Evolutionary Genetics Analysis version 11. *Mol. Biol. Evol.* **38**, 3022–3027 (2021). 33892491 10.1093/molbev/msab120 PMC8233496

